# Embryo versus endometrial receptivity: untangling a complex debate

**DOI:** 10.3389/fendo.2025.1537847

**Published:** 2025-01-22

**Authors:** Ramazan Mercan, Yilmaz Guzel, İrem Usta, Ebru Alper

**Affiliations:** ^1^ Department of Obstetrics and Gynecology, Koc University, Istanbul, Türkiye; ^2^ Department of Obstetrics and Gynecology, Okan University, Istanbul, Türkiye; ^3^ Department of Obstetrics and Gynecology, American Hospital of Istanbul, Istanbul, Türkiye

**Keywords:** implantation, embryo, endometrial receptivity, preimplantation genetic testing, embryo mosaicism

## Introduction

Despite advancements in reproductive technology, the efficiency of assisted human reproduction remains notably low, with clinical pregnancy rates per transfer hovering around 34%, as per the latest report from the European Society of Human Reproduction and Embryology (ESHRE) on IVF monitoring (1).

Efforts to boost pregnancy rates have led to divergent approaches. Some studies focus on embryonal factors, while others aim to enhance endometrial receptivity. Various add-on treatments have been introduced in attempts to enhance the quality of oocytes, sperm, fertilization, embryos, or embryo selection. These include artificial oocyte activation, mitochondrial replacement therapy, sperm DNA damage testing, artificial sperm activation, the sperm hyaluronic acid binding assay (PICSI), Magnetic-activated cell sorting (MACS), intracytoplasmic morphologic sperm injection (IMSI), growth factor-supplemented embryo culture, and the most common add-ons for embryo selection are preimplantation genetic testing for aneuploidy (PGT-A), non-invasive PGT-A, and time-lapse imaging of embryos. However, none of these interventions have proven to be consistently effective in improving outcomes. Moreover, there is limited clear evidence supporting the efficacy of adjuvant growth hormone for ovulation induction, antioxidants, metformin, and Coenzyme Q10 in improving IVF outcomes (2). It is still not possible to explain why %40-45 of euploid embryos does not implant (3).

Numerous studies have been conducted to enhance endometrial receptivity using immunotherapies, corticosteroids, aspirin, heparin, sildenafil, indomethacin, intravenous immunoglobulin, Granulocyte-Colony Stimulating Factor (G-CSF), intralipid, peripheral blood mononuclear cells (PBMC) infusion, intrauterine administration of human chorionic gonadotropin (hCG), hyaluronic acid addition to transfer media, endometrial scratching, intrauterine platelet rich plasma (PRP) administration, freeze-all embryo strategies, and endometrial receptivity array (ERA). However, there is a lack of robust evidence supporting their effectiveness in improving IVF outcomes (3).

## Embryo or endometrial receptivity: which comes first?

The ongoing debate centers around the respective roles of the embryo and endometrial receptivity in ART. The purpose of this review is not to examine all studies on IVF, endometrial receptivity, or embryo quality and selection, but rather to review the latest data on the most commonly used add-ons and to highlight how difficult it is to determine the relative roles of embryo quality and receptivity on IVF outcomes.

Numerous studies have focused on endometrial receptivity. Two-thirds of implantation failures are attributed to endometrial receptivity (4), though there is no scientific evidence clearly distinguishing the relative contribution of embryo quality versus endometrial receptivity to ART outcomes.

The most frequently used add-on treatments to increase endometrial receptivity are supplementation of additional progesterone in patients with low progesterone levels prior to embryo transfer, endometrial scratching, platelet-rich plasma and ERA.

Low progesterone levels on the day of embryo transfer in frozen embryo transfer (FET) cycles have been associated with lower ongoing pregnancy rates. Labarta et al. demonstrated that increasing progesterone doses in patients with levels below 10 ng/mL effectively achieved live birth rates comparable to those with progesterone levels above 10 ng/mL. Notably, regression analysis in that study revealed that low progesterone levels did not impact live birth rates (LBR) after adjusting for confounding factors. A significant difference was observed only when comparing patients who received additional progesterone with a historical control group (5).

In another retrospective cohort study involving 694 patients, similar LBRs were observed between patients with normal progesterone levels (>8.8 ng/mL) and those with lower levels (<8.8 ng/mL) when dydrogesterone was added for luteal support (37.8% vs. 38.8%). Low progesterone levels were identified in 21.2% of patients (6).

In two other studies, both high and low progesterone levels were associated with lower pregnancy rates. Thomsen et al., in a multicenter prospective cohort, found that the optimal chance of pregnancy was achieved with serum progesterone levels of 60–100 nmol/L in the early luteal phase, while optimal levels during the mid-luteal phase ranged from 150–250 nmol/L. The positive hCG rate was 73% in patients with early luteal progesterone levels of 60–100 nmol/L, compared to 35% in those with progesterone levels above 400 nmol/L following cleavage-stage embryo transfer. The optimal progesterone level for LBR was found to be 150–250 nmol/L, yielding an LBR of 54%, compared to 38% in patients with progesterone levels above 400 nmol/L (7). Similarly, Yovich et al. showed that progesterone levels lower than 50 nmol/L and higher than 99 nmol/L were associated with lower implantation rates (8).

In contrast to these findings, Alvarez et al. observed that, following euploid embryo transfer in FET cycles, additional progesterone supplementation in patients with progesterone (P4) levels below 10.6 ng/mL on the day before embryo transfer resulted in comparable pregnancy, ongoing pregnancy, live birth, and miscarriage rates (9).

Similarly, Aslih et al., in a prospective randomized controlled study involving 146 patients, found that increasing progesterone levels 7 days after embryo transfer in patients with P4 levels lower than 15 ng/mL did not result in higher pregnancy rates (10). Finally, a recent study found that progesterone levels on the day of embryo transfer in true natural cycle euploid FET cycles did not differ between patients with and without ongoing pregnancies (11).

In summary, the supplementation of additional progesterone remains controversial. Randomized controlled trials are needed to clarify the role of rescue progesterone in IVF outcomes. Moreover, inter- and intra-assay differences in progesterone levels make it challenging to draw definitive conclusions regarding the role of increasing progesterone supplementation. Additionally, progesterone levels can vary based on factors such as time of day, BMI, parity, and geographic origin.

Platelet-rich plasma (PRP) induces proliferation, angiogenesis, and possesses anti-inflammatory effects. It is prepared by centrifuging peripheral blood, resulting in a concentrated enrichment of platelets. PRP is classified based on its platelet concentration, as well as its leukocyte and fibrin content. Due to its anti-inflammatory, angiogenic, and extracellular remodeling properties, along with its ability to enhance stem cell recruitment, PRP is widely utilized in regenerative medicine, particularly in plastic surgery, dermatology, and orthopedic surgery.

In the context of female infertility, PRP has been applied in patients with refractory thin endometrium, Asherman syndrome, chronic endometritis, and recurrent implantation failure. Ovarian PRP has also been used for poor responders and those with premature ovarian failure.

Platelet-rich plasma (PRP) induces proliferation, angiogenesis, and possesses anti-inflammatory effects. It is prepared by centrifuging peripheral blood, resulting in a concentrated enrichment of platelets. PRP is classified based on its platelet concentration, as well as its leukocyte and fibrin content. Due to its anti-inflammatory, angiogenic, and extracellular remodeling properties, along with its ability to enhance stem cell recruitment, PRP is widely utilized in regenerative medicine, particularly in plastic surgery, dermatology, and orthopedic surgery.

In the context of female infertility, PRP has been applied in patients with refractory thin endometrium, Asherman syndrome, chronic endometritis, and recurrent implantation failure. Ovarian PRP has also been used for poor responders and those with premature ovarian failure.

However, the literature presents controversial findings regarding the effectiveness of PRP on ART outcomes, particularly in patients with thin endometrium. A few non-randomized small studies (12, 13) and two randomized studies (14, 15) have shown a positive impact of PRP on endometrial thickness, implantation, clinical pregnancy, and live birth rates. Conversely, other studies have not confirmed these findings (16, 17). Similarly, in patients with recurrent implantation failure, findings have been mixed. While prospective studies have reported controversial results (18–23), all randomized studies have shown a positive impact of PRP in these patients (24–28). However, the studies are small, heterogeneous, and lack standardization in preparation methods, dosage, and administration routes.

The most recent Cochrane review concluded that the effect of PRP on ART outcomes remains uncertain. The review highlighted several limitations in the available studies, including a high risk of bias, poor reporting methods, lack of prospective registration, and insufficient data. Additionally, some studies failed to report live birth rates (29).

In summary, the role of platelet-rich plasma in enhancing endometrial receptivity remains uncertain, and there is a need for larger, randomized studies to clarify its effects.

The role of endometrial scratching remains controversial. While several studies have shown a positive impact of endometrial scratching prior to IVF, two recent, well-designed randomized controlled trials have found no beneficial effect on outcome measures (30, 31). A recent meta-analysis suggested a positive effect of endometrial scratching on IVF outcomes (32), but it has been criticized for methodological and statistical flaws (33, 34). In a recent randomized controlled study of 124 oocyte recipients, hysteroscopic endometrial fundal incision was associated with a significantly higher rate of positive pregnancy tests (79% vs. 59.7%). However, there was no significant difference in the live birth rate (58.1% vs. 51.6%) (35).

In another prospective study of 109 patients undergoing oocyte donation after a negative first embryo transfer cycle, diagnostic hysteroscopy and endometrial fundal incision were performed in 50 of these patients. Both the positive pregnancy test rate and the live birth rate were significantly higher in the endometrial fundal incision group (36).

In summary, the role of endometrial scratch should be further investigated in randomized trials, both for patients undergoing their first embryo transfer cycle and for those with a previous negative embryo transfer. In addition, alternative approaches such as endometrial fundal incision should be evaluated in larger randomized studies.

Although initial studies with Endometrial Receptivity Analysis (ERA) showed promising results, more recent studies have indicated that ERA may not be effective and, in some cases, could even be detrimental to pregnancy rates (37).

Stem cell or exosomal treatments are still in the early stages of development. Case reports and small studies have shown positive effects, particularly in patients with intrauterine adhesions. However, it is too early to draw definitive conclusions regarding their impact on outcomes (38–40).

Several embryo selection methods have been introduced to enhance ART outcomes, including PGT-A, non-invasive PGT-A (niPGT-A), and time-lapse monitoring, in addition to traditional morphological criteria. However, the effectiveness of PGT-A in improving ART outcomes remains inconclusive. Three randomized studies and recent SART data indicate that PGT-A does not significantly improve IVF outcomes (41–44).

Non-invasive PGT-A (niPGT-A), which analyzes DNA in spent culture media or blastocele fluid, is promising due to its non-invasive nature (45). niPGT-A demonstrates improved accuracy for sex determination. In addition, niPGT-A yields better results when conducted on day 5 blastocysts, rather than on day 3 embryos. Although the overall role of PGT-A in embryo selection is still under debate, most studies investigating niPGT-A have compared the rates of euploidy or aneuploidy against those obtained via conventional PGT-A. Reported concordance rates range from 45.5% to 93.8%, with sensitivity between 33% and 100%, specificity from 48.3% to 87.5%, a positive predictive value (PPV) of 20% to 91.7%, and a negative predictive value (NPV) of 33% to 100%.

In another study, Fang et al. noted a 60% PPV for non-invasive prenatal testing (niPT) in predicting live birth, pointing to a potentially promising role for niPT in the future (46). Nonetheless, key limitations of niPGT-A include DNA amplification failure, maternal DNA contamination, and diagnostic accuracy challenges, greater standardization and reliability are still needed.

The role of time-lapse systems (TLS) in embryo incubation and their impact on IVF outcomes remains unclear. Two recent multicenter randomized controlled studies found that the use of time-lapse imaging for embryo culture and selection does not significantly increase the live birth rate (47, 48).

On the other hand, some researchers have proposed that the embryo plays the most significant role in implantation. Assuming that PGT-A represents the most significant and effective advancement in embryo selection and that the endometrium plays a minimal role, a new definition for recurrent implantation failure has been proposed. According to this newer perspective, the endometrium acts merely as a receptive organ or is responsible for less than 5% of implantation failure.

Pirtea et al. have reported an impressive pregnancy rate of 95% following three euploid embryo transfers, suggesting that embryo aneuploidy may indeed be a significant determinant of implantation success (49). Nonetheless, several limitations need to be acknowledged in this study. Firstly, the study cohort does not adequately represent a typical IVF population. It comprises a highly selective group characterized by a mean age of around 35 years, a BMI of 25, an average of 12 retrieved oocytes, and approximately 3.5 euploid embryos per patient. The age range spans from 18 to 45 years, with the lowest AMH level recorded at 3 ng/ml. It’s worth noting that in a standard IVF population, encountering a 45-year-old patient with a 3 ng/ml AMH level and an average of 3.5 euploid embryos would be rare. Furthermore, all patients in this study possessed anatomically normal uterus with a minimum endometrial thickness of 7 mm. Therefore, extrapolating a new definition of recurrent implantation failure from this highly selective and favorable prognosis group might not be appropriate. Additionally, drawing conclusions that achieving a 95% pregnancy rate after three consecutive cycles is universally achievable could be misleading. Instead, it would be more prudent to provide information based on pregnancy rates per cycle and estimate the number of cycles required to achieve optimal outcomes, considering factors such as age and ovarian reserve markers. In cases where obtaining even one euploid embryo within multiple cycles may be unrealistic, particularly for patients over 40, providing tailored information considering individual circumstances becomes paramount.

Secondly, the attempt to establish a novel definition of recurrent implantation failure based on euploid embryo transfer lacks a control group of patients undergoing untested or mosaic embryo transfers. Despite some drawbacks, several randomized controlled trials have demonstrated no discernible difference in cumulative pregnancy rates between euploid and untested embryo transfers (3, 41–44). Hence, the utility of the proposed new definition of recurrent implantation failure, centered on PGT-A for euploid embryo transfer, remains contentious and not yet a standardized procedure in routine IVF practice.

Another limitation of the study lies in its considerable patient dropout rate. Although the authors noted that dropout patients typically lacked remaining embryos, suggesting a relatively poor prognosis group, they reported no demographic differences between patients in the first and third cycles.

In another study by Polyzos et al., a cumulative live birth rate (CLBR) of 60-70% was reported when more than 25 oocytes were retrieved (50). While this study did not involve patients undergoing PGT-A, it is reasonable to expect that such patients would possess at least 3.5 euploid embryos. This CLBR stands substantially lower than the rates documented by Pirtea et al. Discrepancies could be attributed to variations in freezing and media conditions across studies, as well as differences in the transfer of untested embryos. Nevertheless, elucidating the variation in CLBR compared to Pirtea et al.’s findings remains challenging.

In a recent study, Almohamady et al. reported a sustained implantation rate of 77.1% and a live birth rate of 68.8% following three successive euploid embryo transfers (51). Intriguingly, they observed implantation failure in 20% of patients after three cycles, contrasting with only 5% in Pirtea et al.’s study. This variance could stem from multiple factors, including disparities in mean age and other demographic characteristics between the patient groups. However, Almohamady et al.’s cohort had a lower mean age compared to Pirtea et al.’s, suggesting that age alone might not account for the difference. Additionally, Almohamady et al.’s patients were also predominantly good responders, characterized by a higher mean number of oocytes and blastocysts biopsied compared to Pirtea et al.’s study.

One of the most significant challenges in preimplantation genetic testing for aneuploidy (PGT-A) is determining whether to transfer mosaic embryos. Reported mosaicism rates in embryos range from 2% to 40% (52). However, the incidence of mosaicism in newborns is reported to be less than 0.2%.

Embryo mosaicism arises post-zygotically due to mitotic errors during the early stages of embryonic development, particularly within the first three cell divisions (53). Mosaicism can be classified using several parameters, including the percentage of aneuploid cells), the number of chromosomes involved and the type of abnormality (whole-chromosome or segmental mosaic) (54). There is currently no consensus on the exact thresholds for low-level or high-level mosaicism; indeed, low-level mosaicism has been reported as anywhere between 20% and 80%.

Although some studies have found that mosaic embryos demonstrate implantation and miscarriage rates comparable to those of euploid embryos, the majority of research points to lower implantation and higher miscarriage rates with mosaic embryos (55–57). Nevertheless, no significant differences in neonatal outcomes have been reported (58). Notably, Lin et al. observed that high-level mosaic embryos exhibited live birth rates similar to those of low-level mosaics but were associated with higher miscarriage rates (59).

A comprehensive review of existing studies suggests that while the live birth rate following the transfer of whole-chromosome aneuploid embryos is 2% or less, the findings regarding putative mosaic embryos are more variable.

The diagnostic accuracy for mosaicism may be influenced by factors such as the biopsy technique, the next-generation sequencing (NGS) platform used, the cutoffs applied for defining mosaicism, the thresholds for data interpretation, and the specific chromosomes involved. Furthermore, the limited number of cells analyzed (often 5–10) may not accurately represent the entire embryo. An apparently aneuploid embryo could still harbor euploid cells, and an embryo classified as euploid could in fact be mosaic (60).

In a multicenter, prospective, blinded, non-selection study, Tiegs et al. observed no significant difference in the sustained implantation rate between embryos subjected to PGT-A and age-matched controls. Notably, none of the aneuploid embryos achieved sustained implantation. Interestingly, 11 out of 16 embryos with whole chromosome mosaicism successfully implanted. These findings highlight the utility of PGT-A in identifying and deselecting aneuploid embryos but raise questions about its effectiveness as a selection tool.

Based on the current evidence, it is not advisable to routinely discard embryos with results in the mosaic range, as excluding these embryos from transfer may have a detrimental impact on the cumulative live birth rate per cycle. Further research is needed to refine diagnostic protocols, establish standardized thresholds, and clarify the prognostic implications of mosaicism in the clinical setting.

In a separate study, Ata et al. found no significant difference in pregnancy rates among patients with endometrial thicknesses exceeding 4 mm (61). However, the retrospective design of the study raises questions about whether embryo transfers were performed irrespective of endometrial thickness or following a canceled cycle with subsequent endometrial evaluation, which may have included beneficial interventions such as endometrial scratching. Additionally, the limited number of patients in the 4-6 mm group makes it challenging to draw definitive conclusions about the role of endometrial thickness or receptivity in IVF outcomes.

While the studies by Pirtea et al. and Ata et al. highlight the crucial role of the embryo in implantation, attributing unsuccessful outcomes solely to embryonic or endometrial factors is challenging due to the potential influence of other confounding variables. To draw definitive conclusions about the role of embryos or endometrial receptivity in implantation failure or pregnancy rates, consistency in one of these factors across all cycles would be necessary. Endometrial receptivity array (ERA) studies have demonstrated variability in receptivity markers across cycles, while previous research has shown that pregnancy rates are influenced by morphological criteria, time to blastocyst stage, and patient age in euploid embryos (62). Additionally, explaining the approximately 25% of cases that did not result in pregnancy in the first cycle, despite selecting the best euploid embryo and having normal uterine anatomy in Pirtea et al.’s study, remains a challenge. Disparities in pregnancy rates can also be attributed to laboratory conditions, light exposure, oocyte handling and manipulation, culture media, and the complexity of the embryo transfer process.

These studies emphasize the importance of having an anatomically normal uterus and a euploid embryo as critical factors for a successful pregnancy. However, they do not address why some patients do not achieve pregnancy in the first cycle despite having a euploid embryo and a normal uterus. Successful pregnancies in subsequent cycles may be due to morphological differences in the embryo or cycle-to-cycle variations in the endometrium.

## Conclusion

In conclusion, it is challenging to definitively determine whether embryonic factors or endometrial receptivity are more crucial for a successful pregnancy. While having an anatomically normal uterus and a euploid embryo are essential for achieving pregnancy, precisely quantifying the role of each is difficult. For clearer conclusions, at least one of these factors would need to remain constant across all embryo transfers. However, we know that not all euploid embryos are identical, and endometrial receptivity can vary from cycle to cycle. Furthermore, none of the current add-ons aimed at improving embryo quality, endometrial receptivity, or embryo selection have proven consistently effective in enhancing ART outcomes.

Artificial intelligence (AI) algorithms, utilizing static or dynamic images of embryos and endometrial organoid models, are emerging as promising tools in reproductive medicine. These algorithms can analyze IVF images to predict embryo viability and implantation potential, identifying subtle patterns and features that may not be visible to the human eye, potentially offering more accurate predictions.

Combining PGT-A with AI algorithms could further improve embryo selection. By assessing both genetic and morphological characteristics of embryos, clinicians may make more informed decisions about which embryos to transfer, thereby increasing the likelihood of a successful pregnancy.
